# Inotropic Agents: Are We Still in the Middle of Nowhere?

**DOI:** 10.3390/jcm13133735

**Published:** 2024-06-26

**Authors:** Anna Maria Iorio, Fabiana Lucà, Andrea Pozzi, Carmelo Massimiliano Rao, Stefania Angela Di Fusco, Furio Colivicchi, Massimo Grimaldi, Fabrizio Oliva, Michele Masssimo Gulizia

**Affiliations:** 1 Cardiology Department, Papa Giovanni XXIII Hospital, 24127 Bergamo, Italy; anita.iorio@hotmail.it; 2 Cardiology Department, Grande Ospedale Metropolitano, 89129 Reggio Calabria, Italy; massimo.rao@libero.it; 3 Cardiology Division, Valduce Hospital, 22100 Como, Italy; andreawellsvabg@gmail.com; 4 Cardiology Department, San Filippo Neri Hospital, ASL Roma 1, 00135 Rome, Italy; doctstefania@hotmail.com (S.A.D.F.); furio.colivicchi@gmail.com (F.C.); 5 Department of Cardiology, General Regional Hospital “F. Miulli”, 70021 Bari, Italy; m.grimaldi@miulli.it; 6 Cardiology Department De Gasperis Cardio Center, Niguarda Hospital, 20162 Milan, Italy; fabri.oliva@gmail.com; 7 Cardiology Department, Garibaldi Nesima Hospital, 95122 Catania, Italy; michele.gulizia60@gmail.com

**Keywords:** inotropic agents, vasoactive drugs, non-adrenergic agents, adrenergic agents, acute heart failure

## Abstract

Inotropes are prescribed to enhance myocardial contractility while vasopressors serve to improve vascular tone. Although these medications remain a life-saving therapy in cardiovascular clinical scenarios with hemodynamic impairment, the paucity of evidence on these drugs makes the choice of the most appropriate vasoactive agent challenging. As such, deep knowledge of their pharmacological and hemodynamic effects becomes crucial to optimizing hemodynamic profile while reducing the potential adverse effects. Given this perspective, it is imperative for cardiologists to possess a comprehensive understanding of the underlying mechanisms governing these agents and to discern optimal strategies for their application across diverse clinical contexts. Thus, we briefly review these agents’ pharmacological and hemodynamic properties and their reasonable clinical applications in cardiovascular settings. Critical interpretation of available data and the opportunities for future investigations are also highlighted.

## 1. Introduction

Positive inotropic drugs can be defined as treatments that enhance myocardial contractile performance without affecting heart rate (HR) or loading conditions. Conversely, vasopressor agents are selectively administered to improve vascular tone [1].

Notwithstanding the documented association with heightened mortality rates, these pharmacological agents constitute an essential therapeutic intervention for individuals in critical conditions exhibiting hemodynamic instability. Their application continues to be advocated for specific patient cohorts displaying indications of reduced cardiac output and peripheral hypoperfusion [2]. However, the identification of specific agents can be challenging.

Indeed, evidence for inotropic and vasopressor therapy has been scarce, and better data are needed to compare the efficacy of such drugs within specific clinical settings. The scenario has remained unchanged over the past two decades, yielding limited reliable data to assist clinicians in selecting the most suitable agent.

Thus, in cardiovascular disease (CVD), the choice of vasoactive strategy should balance the hemodynamic effect and assess hemodynamic goals. The effectiveness of these agents should be further evaluated based on their tendency to increase adverse events. In this regard, fundamental insights into these medications regarding their pharmacological and hemodynamic effects within various cardiovascular (CV) clinical contexts remain pivotal.

Although several reviews have been reported [2,3], this review provides physicians with an update on these drugs’ main pharmacological and hemodynamic effects and reasonable clinical applications in CV settings. We also highlight critical interpretation of available data and opportunities for future investigations.

## 2. Use of Inotropes and Vasopressors in Clinical Practice

The administration of vasoactive agents has been practically unchanged in the last decades as the drugs continue to be used in acute heart failure (AHF) patients with the same prevalence [4].

Indeed, in clinical practice, the use of inotropes and vasopressors often remains inappropriate. Table 1 summarizes the characteristics of treated patients across different regions. Of note, the most frequent clinical scenario was decompensated HF. However, many patients showed no signs of hypoperfusion or low cardiac output. A systolic blood pressure > 110 mmHg and a left ventricular ejection fraction (LVEF) > 30% were present in almost 50% of patients in the IN-CHF registry [5]. Similarly, the ADHERE registry showed a mean systolic blood pressure of 144 mmHg and a mean LVEF of 38% in patients treated with active agents [6].

It is also interesting to note that the use of specific agents remains rather heterogeneous. As reported in Table 1, the administration of single agents differs across regions, highlighting that the choice of these drugs appears to be related to local clinical practice. In the Italian registry, dopamine (71%) was the most frequent inotrope, followed by dobutamine (40%) and levosimendan (20%) [5]. In contrast, in the American registry, dobutamine was the most used agent, followed by dopamine and levosimendan [6] (Table 1).

## 3. Exploring the Evidence through Trials and Registry: An Issue of Concern

The administration of vasoactive agents has been linked with elevated mortality rates. However, several considerations are needed to elucidate their effects on survival. Primarily, the utilization of inotropes/vasopressors is typically restricted to profoundly unwell patients. Thus, the observed association with heightened mortality may be merely coincidental. This dilemma underscores one of the most intricate challenges in deciphering the potential cause-and-effect relationship between these pharmaceutical interventions and clinical outcomes [11].

Further, large randomized placebo-controlled trials (RCT) are difficult to perform, resulting in often being unpowered, wherein heterogeneous populations with multiple physiopathologic pathways and different goals are often included [12]. In particular, previous analyses have not been adequately powered to assess the comparative effectiveness of vasoactive agents, leading to an important knowledge gap for the most appropriate choice of these medications.

Considering the above issues of RTC, statistical tests have been developed to mitigate the limitations of observational data. However, the complexity of statistical tests is beyond the understanding of most clinicians, and some results are not easily interpretable. In this sense, as Mortara underlined, careful attention is required when the results of the propensity score analysis are interpreted [13]. Indeed, a propensity-based analysis may exclude patients with more severe hemodynamic impairments, thus ruling out patients who are most frequently treated with these drugs. Another limitation characterizing the interpretation of observational data analysis is related to the scarcity of knowledge regarding the vasoactive administrated dosage that, in turn, might induce detrimental effects when used at high doses or limit a hemodynamic response when used at low fixed doses.

Notwithstanding the aforementioned critical insights, all available data indicate that acting drugs are harmful in the short term, especially when used in the absence of signs of cardiogenic shock (CS) [13,14,15,16,17]. Thus, international guidelines restrict the use of these drugs (level of evidence C) in HF patients with signs of hypoperfusion and/or in patients presenting CS [18,19]

## 4. Adrenergic Agents

Most intravenous vasoactive drugs exert their hemodynamic effects by enhancing the adrenergic pathway [20], increasing contractility and heart rate through cardiac β1-receptors and vasoconstriction via peripheral α-receptors. Additionally, some inotropes reduce afterload from systemic vasodilation by β2-receptors. However, these drugs do not display a “pure” pharmacological profile. Indeed, their actions are mediated by the stimulation of different adrenoceptors conferring a hemodynamic effect that depends on the receptor localization, drug–receptor affinity, and dose administered, determining a large spectrum of hemodynamic pattern responses to circulation (vasodilation or vasoconstriction), cardiac output (inotropic effect), and myocardial relaxation (lusitropic effect) [20]. Notably, these drugs exhibit a dose-dependent hemodynamic action, wherein the most common detrimental effects occur at high doses. Table 2 summarizes their key pharmacologic and hemodynamic properties.

Adrenaline (epinephrine) is a potent agonist of adrenoceptors, resulting in a profound increase in cardiac output, mean arterial pressure, and coronary blood flow [1]. At low doses, this agent may accommodate the passive stretch of pulmonary vessels, whereas at high doses, it may increase pulmonary vascular resistance (PVR) and right ventricular (RV) afterload [14,21]. The exact toxic mechanisms of epinephrine remain unclear. However, increased oxygen consumption and altered calcium homeostasis appear to be the main deleterious effects on the myocardium [14,21]. This is especially relevant in patients with CS caused by acute coronary syndrome (ACS), in whom adrenaline use was associated with a significantly increased risk of mortality. In this regard, a recent randomized clinical trial that included patients with CS often affected by ACS (80% of enrolled patients) showed that adrenaline administration was associated with safety concerns when compared with other vasopressors [15,22], emphasizing the particularly detrimental effects of adrenaline in this setting. However, it must also be noted that adrenaline was administered at high doses (maximum dose 0.22 mcg/kg/min), and most adrenaline-treated patients often also received a high dose of noradrenaline or dopamine that may have contributed to severe organ function impairment. Furthermore, this study’s small size and the long enrollment duration require further studies to confirm this finding.

In observational prospective analysis, including unselected CS patients, adrenaline was also associated with higher mortality when compared with other vasopressors/inotropic regimens [16,23]. Likewise, the lack of information about doses and treatment duration makes these results only explorative.

Further trials demonstrating safety and efficacy are required. Particularly, in an era of heightened efforts to mitigate the untoward effects of vasoactive drugs, future studies exploring adrenaline administration at low to medium doses should be encouraged.

Noradrenaline (norepinephrine) is often described as a pure vasopressor because of its marked α-adrenoceptor and a weak β- adrenoceptor effect [1]. Its α –receptors-mediated vasoconstriction effect is helpful in patients with CS. Activation of these receptors also causes pulmonary vasoconstriction, although the effects on PVR occur more frequently at high doses [24]. Compared with adrenaline, however, the pulmonary vasoconstrictive effect of noradrenaline may be more harmful due to its weak inotropic effect.

Considering its mechanism of action on PVR and its poor effect on myocardial contractile performance, noradrenaline is commonly [15] associated with inotropic/vasodilator agents (e.g., dobutamine or levosimendan) in the subset of patients with CS [25]. Interestingly, this combined approach was not associated with excessive mortality when compared with other vasopressor/inotropic regimens [25]. However, as mentioned above, future studies are encouraged to address this comparative efficacy.

Dopamine, an endogenous neurotransmitter, is an immediate precursor of norepinephrine in the catecholamine synthetic pathway. It acts on both dopaminergic (D1) receptors and adrenoceptors and is characterized by a complex CV response profile with predominantly dose-dependent effects [1]. At high doses (>5 μg/kg/min), it activates α1 and α2 receptors, producing vasoconstriction. At intermediate doses (3–5 μg/kg/min), it activates β1 receptors with predominantly inotropic effects. At low doses (<3 mg/kg/min^−1^), it increases renal blood flow. Of note, detrimental effects such as ventricular and supraventricular arrhythmias at high doses have limited its use as an inotropic/vasoconstrictive agent [26]. However, the renal effect of dopamine has led to huge speculation regarding its use across different spectrums of HF patients. In support of a low renal dose effect, a previous study assessed the hemodynamic renal effect of dopamine with an intravascular Doppler [27], showing that dopamine effectively increased renal blood flow at a low dose. However, contradictory results regarding the true benefit of this approach have been reported [28,29]. Metra et al. found high inter-patient variability in the dose response of dopamine for increasing renal blood flow [21,30], suggesting that a dose treatment should be individualized. The Renal Optimization Strategies Evaluation in AHF (ROSE AHF) trial [28] showed no benefit in using a low fixed dose of dopamine in patients with AHF and renal dysfunction. This failure may be partially attributed to the heterogeneity of enrolled patients with HF, along with the fixed dose used (i.e., not individualized).

However, in line with previous suggestions that inotropic effects may also occur at low doses, a post hoc analysis of the ROSE AHF trial showed a renal function improvement in patients with LVEF < 40% [31]. Hence, all of the above insights may suggest that dopamine in AHF should be individualized and reserved mainly for hypotensive HF patients with reduced LVEF [29,32].

Dobutamine is a synthetic catecholamine with mixed β-adrenoceptor effects. It binds in a 3:1 ratio to β1 and β-2 adrenoceptors, respectively. At low doses, dobutamine increases myocardial contractility and reduces PVR, improving ventricular–arterial coupling [33]. However, high doses (>10 μg/kg/min) lead to an increased risk of myocardial ischemia and arrhythmias [34,35].

Its long-acting pharmacokinetic properties on vascular endothelial function have led to a home- or outpatient-based continuous infusion in patients with advanced chronic HF [36,37,38]. Despite its hemodynamic effects, the previous randomized study revealed that patients treated with continuous dobutamine infusion may have a higher risk of adverse events such as resuscitated arrest, myocardial infarction, and death [39]. Thus, their routine use should be considered in selected patients believed to be “inotrope-dependent” [40].

Although its pharmacologic properties are salient to achieve the appropriate therapeutic goals, it is important to be aware of its marked variability in treatment response. Indeed, since dobutamine exerts its effects by stimulating β1 adrenergic receptors, its action may decrease in the failing heart, characterized by myocardial β downregulation [41]. Additionally, pharmacodynamic tolerance was observed during long infusion [42].

Combined treatment with other vasoactive medications may optimize its hemodynamic profile while reducing potential adverse effects. In this regard, dobutamine with dopamine is effective in reducing pulmonary wedge pressure and right ventricular end-diastolic pressure while increasing stroke volume with a lower arrhythmogenic profile [43]. Similarly, Nanas et al. reported that combined therapy with dobutamine and levosimendan in decompensated HF patients was associated with increased cardiac index and decreased pulmonary wedge pressure [44]. All these data have unanimously suggested combination therapy as a reliable and safe approach to achieve a hemodynamic effect with a low rate of adverse effects.

## 5. Non-Adrenergic Agents

Inotropic agents acting on non-catecholamine pathways have been developed, leading to positive inotropy without increasing myocardial oxygen consumption [1].

Phosphodiesterase III inhibitors (PDEI3) increase cAMP levels in cardiomyocytes by inhibiting AMP breakdown by the sarcoplasmic reticulum-associated enzyme phosphodiesterase III [45]. The cAMP concentration increases calcium influx into the cell, increasing contractility. Furthermore, these drugs induce peripheral and pulmonary vasodilation by inhibiting PDEI3 in vascular smooth muscle cells, consequently reducing systemic arterial, pulmonary arterial, and venous pressure. Considering their positive inotropic and vasodilatory effects, these agents are frequently called “inodilators”.

Of note, PDE3 enhances the β-receptor-mediated pathway irrespective of the β-receptor, overcoming the desensitization and downregulation of cardiac β-receptors, with an increased inotropic and lusitropic effect in advanced HF patients chronically treated with beta-blockers [46].

The reasonable use of PDE3 inhibitors has also been shown in patients with RV failure and pulmonary vascular dysfunction. Both milrinone and enoximone have been found to increase RV function and decrease PVR, improving RV/pulmonary coupling. However, due to the high risk of systemic hypotension, co-administration with a vasopressor is often needed [47].

Despite their hemodynamic benefits, enhancing drug-mediated intracellular cAMP may trigger maladaptive cardiac remodeling, increasing the risk of ventricular arrhythmias [48]. Importantly, careful attention should be paid to patients with coronary heart disease since the use of milrinone has been associated with worse outcomes [49].

Levosimendan is a Ca^2+^ sensitizer and exerts its inotropic action by enhancing the sensitivity of troponin-C to intracellular Ca^2+^ [50]. Its effect increases the myocardial force at any given cytosolic Ca^2+^ concentration, shifting the relationship between cytosolic Ca^2+^ concentration and the force development of sarcomeres to the left.

Levosimendan also exerts a synergic inotropic effect by selective PDE3 inhibition, a mechanism that is particularly relevant when endogenous or exogenous catecholamines activate beta-adrenoceptors. Furthermore, since it can open ATP-sensitive potassium channels in vascular smooth muscle cells, it acts on arteriolar and venous dilatation, modifying critical pathways in HF pathophysiology [1]. Thus, careful attention should be given to hypotensive patients. Accordingly, levosimendan should be initiated with a variable infusion rate according to systemic pressure, from 0.05 to 0.2 μg/kg/min [48,49,50,51].

Its pharmacokinetic effects are characterized by a relatively short half-life (1 h), but its active metabolite has a much longer half-life (81 h) with a peak concentration between 48 and 72 h [52]. These pharmacokinetic properties and the limited pro-arrhythmogenic and energy-consuming effects make this drug ideal in different clinical HF scenarios. In advanced HF patients, intermittent repetitive administration has been suggested as a reliable strategy [48,49,50,51]. Although this approach has not been associated with survival improvement, data from randomized clinical trials and observational studies have shown reduced NT-proBNP values and hospital admissions [52].

Of note, levosimendan is useful as a predictive tool to detect left ventricular assist device dysfunction in right-sided HF [42,51]. Sponga et al. found that patients with improved cardiac index, pulmonary artery pressure, and central venous pressure after levosimendan administration were less likely to develop post-operative right-sided HF [45,53,54]. Considering the importance of this clinical issue, further studies are needed to confirm these data.

Among different AHF scenarios, levosimendan appears promising in HF associated with ACS since it seems to preserve and enhance myocardial function without increasing oxygen consumption [42,51]. The RUSSLAN trial, which was designed to address safety concerns about levosimendan in patients with HF and ACS, found that levosimendan was associated with a reduction in HF worsening and death [55].

## 6. Other Inotropes

Recently, a new inotropic agent with a safety profile has been studied to improve cardiac performance in both chronic and acute settings. Indeed, novel agents with different mechanisms of action have been proposed. Omecamtiv mecarbil, pimobendane, and istaroxime appear to be potential treatment opportunities in patients with HFrEF.

Omecamtiv Mecarbil is a selective cardiac myosin activator. This drug stabilizes the myosin lever arm in a primed position, accumulating cardiac myosin head in a primed pre-powerstroke state before the onset of cardiac contraction [56]. In this regard, omecamtiv meracabil increases the number of myosin heads bound to the actin, enhancing cardiac contractility. As a result, this inotrope increases the cardiomyocytes contractility by the direct action of the myofilament without changes in the calcium transient [56,57]. The GALACTIC-HF trial was designed with the hypothesis that omecamtiv mecarbil can safely improve symptoms, prevent clinical HF events, and delay CV death in patients with chronic HF. With these aims, omecamtiv mecarbil reduced the primary endpoint of the first HF event or CV death [58,59]. In the GALACTIC-HF trial, a cardiac inotrope demonstrated a safety profile, and no difference in the adverse event (i.e., ventricular tachycardia, myocardial infarction) compared with placebo emerged. However, when the secondary endpoint of CVdeath was considered, omecamtiv mecarbil did not show any difference compared with placebo. Although omecamtiv mecarbil seems a promising inotrope to treat patients with HFrEF, further studies are needed to investigate its role in acute settings with hemodynamic impairment.

Pimobendan has cardiotonic vasodilation properties derived from the combination of phosphodiesterase-inhibiting and calcium-sensitizing inotropic agents. It was tested in the late 1990s in patients with HFrEF. However, in the only randomized controlled trial assessing the effect of this drug on mortality [60], oral administration of pimobendan was associated with 12% of deaths compared with 5.6% in the placebo arm. Consequently, the clinical development of pimobenad was discontinued, although it remains clinically approved in Japan only.

Istaroxime is a novel pharmacologic agent with both inotropic and lusitropic properties. This drug has been shown to reduce pulmonary wedge pressure, improve diastolic function, increase cardiac index in patients with HF at high doses, and reduce ejection fraction [60]. However, according to the ESC heart failure guidelines, this drug has been tested in patients with no indication of inotropic drugs since patients had a systolic blood pressure of 150–90 mmHg.

## 7. Using Inotropic/Vasopressor Agents across Different Clinical Settings

Distinguishing between different clinical entities based on physiological characteristics and the underlying etiology of hemodynamic instability is crucial for administering effective therapeutic strategies. However, the lack of comprehensive data in this area poses challenges. Even when positive statistical results are obtained, caution is warranted due to the high heterogeneity among trial patients [61] and the absence of tested protocols for individualized treatment. In the absence of robust evidence, physicians should base the right agent selection on interpreting patient physiology while incorporating the hemodynamic effects of drugs, along with potentially relevant information derived from observational data. By carefully considering all these factors, physicians can make informed decisions customized to meet each patient’s unique needs (Table 2). In acute HF with LV dysfunction and low cardiac output, dobutamine remains a commonly used first-line choice in real-world clinical practice [61]. However, alternative agents such as levosimendan and phosphodiesterase inhibitors may be preferred over dobutamine in certain situations. This preference may arise in patients who are already on beta-blocker therapy or in clinical scenarios where a vasodilator effect is needed [61]. Of note, particular attention might be required when acute-on-chronic heart failure HF emerges. Indeed, patients with long-standing HF undergo significant physiological adaptations that improve their tolerance to low cardiac output states and/or elevated ventricular filling pressures [62,63]. A retrospective analysis of the heart failure-related cardiogenic shock Critical Care Cardiology Trials Network (CCCTN) registry [64] revealed higher overall mean pulmonary arterial pressures and lower median pulmonary arterial pulsatility indices in cases of acute-on-chronic HF-CS compared with de novo HF-CS. Similarly, Lim et al. demonstrated that patients experiencing HF-CS exhibit a distinct phenotype compared with those with acute myocardial infarction (AMI), characterized by elevated filling and pulmonary artery pressures [65]. Ongoing, Bertaina M et al. concluded that a higher congestive profile (higher CVP at index evaluation) and worsened biventricular dysfunction [66] appear more characterizing patients with CS-HF than patients with CS from AMI. Together, these findings advocate for strategies to enhance cardiac output while mitigating factors that could elevate pulmonary resistance in patients with chronic HF. From this perspective, certain vasoactive agents that induce favorable ventricular–arterial uncoupling by combining inotropic properties with pulmonary vasodilation should be considered (see Table 3). When the clinical trajectory of chronic HF necessitates vasopressor agents, high doses of noradrenaline and dopamine may elicit deleterious effects, while low doses of adrenaline may yield a more favorable hemodynamic response [67,68]. On the contrary, patients experiencing hemodynamic deterioration due to AMI may require a treatment strategy aimed at optimizing cardiac output while simultaneously minimizing myocardial oxygen consumption and enhancing coronary flow [67]. Additionally, it appears that low systemic vascular resistance (SVR) is more strongly associated with CS-AMI compared with other clinical scenarios. Collectively, these findings may partially elucidate the superior efficacy of noradrenaline over other vasopressor strategies in patients experiencing CS-AMI. Recent attention has been focused on managing right ventricular failure (RVF). The selection of positive inotropic/vasopressor therapy should be carefully guided by an understanding of the primary pathophysiological mechanisms underlying RVF and the clinical context in which RVF occurs. Ideal treatment strategies would consider systemic arterial pressure to ensure sufficient right coronary filling pressure and RV contractility while avoiding exacerbation of pulmonary artery/RV coupling. To counter the decline in systemic arterial pressure in the context of RVF, vasopressors may be used alongside inotropic or inodilator agents to improve RV/pulmonary coupling. In this perspective, administering low-dose adrenaline can effectively enhance vascular tone and RV contractility with minimal impact on pulmonary vascular resistance (PVR). Conversely, high doses of α1-agonists should be avoided as they can constrict pulmonary vessels, potentially exacerbating RV afterload. Likewise, to address pulmonary hypertension-associated right ventricular failure (PH-RVF) in patients with elevated PVR and hypotension, combining an inodilator with a vasopressor agent is a plausible strategy to reduce PVR while maintaining mean arterial pressure (MAP). For this purpose, low-dose adrenaline has been shown to synergize effectively with milrinone) [20]. In the context of ADHF, RVF can also occur, necessitating a focus on reducing excessive afterload as the primary means of improving RV function. Once again, in this scenario, the hemodynamic goals involve increasing contractility through inotropic agents while concurrently reducing pulmonary vascular tone (see Table 3). However, in cases of advanced ADHF, interactions between the RV and LV may result in the LV becoming dependent on atrial contraction for filling, making it less tolerant of excessive vasodilation. In such cases, the selection of an inotropic agent with specific arrhythmic and vasodilator properties should be approached with particular caution. The primary goal is to enhance RV contractility when RVF arises in the context of normal pulmonary vascular resistance, such as in myocardial infarction. In such situations, low-dose dopamine represents a reasonable option as it can increase cardiac output without adversely affecting PVR. However, caution should be exercised with higher doses of dopamine as they may lead to unwanted β2-mediated vasodilation. Similarly, while low doses of dobutamine can improve myocardial contractility, higher doses should be avoided due to the risk of β2-mediated vasodilation (see Table 3).

## 8. Reasonable Approach and Conclusions

Although the choice of vasopressors or inotropes is still complex, careful selection of vasoactive medications remains widely based on knowledge of the pharmacological mechanisms of such drugs, along with the pathophysiology characterizing the hemodynamic instability to optimize clinical profile while reducing the potential adverse effects. The increase in PVR may play a central adverse role among hemodynamic mechanisms, especially in those with advanced HF. As such, an important effort should be directed at mitigating any effects that may increase PVR. In this sense, certain vasoactive agents, alone or in combination, should favorably yield ventricular–arterial uncoupling, combining inotropic properties and pulmonary vasodilatation [43].

Further, it can also be noted that most cardiac and non-CV adverse effects are widely related to high doses of inotropes and vasopressors. In particular, excessive α-receptors stimulation produces coronary artery vasoconstriction, leading to myocardial ischemia [57] or impairment in splanchnic and renal perfusion with a consequent organ injury [69]. In contrast, excessive cardiac β1R/β2R stimulation predisposes patients to atrial and ventricular arrhythmias [59,70].

An individualized regimen with increasing and decreasing dosages according to the circulatory response should be preferred over a fixed-dose regimen. Furthermore, combination therapy may be preferable to a single-agent high-dose strategy [71,72,73]. Particularly, the combination of a predominant α-adrenergic agent (i.e., norepinephrine) with a β1-adrenergic agonist (i.e., dobutamine) or non-adrenergic agents may selectively titrate vasoactive and inotropic hemodynamic effects [72,74,75]. This could translate to a more favorable hemodynamic effect by achieving a good balance between increased vascular systemic resistances and tissue blood flow, with lower toxic effects related to high dosages of inotropic/vasoactive agents [72].

Furthermore, the parallel administration of vasoactive adrenergic agents with vasodilator non-adrenergic agents may improve ventricular–arterial coupling and maintain adequate mean arterial pressure. However, the evidence supporting this concept is missing.

Finally, although pharmacological properties are salient to achieve the appropriate therapeutic goals, special attention should be given to the marked interindividual variability in treatment response. This variability in treatment response suggests a potential role of other factors in influencing drug–receptor interactions [76]. Thus, the effectiveness of vasoactive agents should be further evaluated based on an individualized regimen.

## Figures and Tables

**Table 1:** Baseline characteristics of patients treated with inotrope across different regions.

	European Registries			American Registry		Multinational Registry
	IN-HF [5] (*n* 360)	ESC-LT [7] (*n* 833)	AHEAD [8] (*n* 4153)	ADHERE [9] (*n* 159,168)		ALARM-HF [10] (*n* 1617)
Region(s)	Italy	Europe	Czech Republic	US		Europe, Australia, Mexico
Years	2007–2009	2011–2013	2006–2009	2001–2003		2006–2007
Age (±sd or IQR)	70 (±12)	67 (±13)	74 (49–87)	72 (±14)		
Male, %	64.4	66	53.6	48.4		65.1
Mean SBP, mmHg (±sd or IQR)	112 (±27)	112 (±27)	135 (85–200)	144 (±33)		100 (85–140)
SBP < 110, *n* (%)	176 (49)	-	-	-		-
SBP ≤ 100, *n* (%)	-	-	648 (15.6)	-		-
SBP > 140, *n* (%)	-	-		79,584 (50)		-
LVEF, % (±sd or IQR)	31 (±12)	35 (±15)	37 (16–65)	37.8 (±17.3)		33.9(±14.2)
LVEF < 30%, *n* (%)	186 (51.6)	-	1574 (37.9)	-		-
LVEF < 40%, *n* (%)	61 (16.9)	-	-	81,653 (51.3)		-
Clinical Setting						
Decompensated HF, *n* (%)	144 (40)	395 (47.4)	224 (53.9)	148,305 (93)		1135 (70.2)
Pulmonary edema, *n* (%)	82 (22.8)	124 (14.9)	748 (18.0)	-		-
RV failure, *n* (%)	29 (8.1)	23 (2.8)	156 (3.7)	-		-
ACS, *n* (%)	67 (18.6)	107 (12.8)	-	6366 (4)		-
Cardiogenic shock, *n* (%)	30 (8.3)	158 (19.0)	600 (14.4)	-		425 (26.3)
Inotrope (%)						
Dopamine, *n* (%)	258 (71.6)	206 (24.7)	352 (8.7)	-		541 (33.5)
Dobutamine, *n* (%)	143 (39.7)	354 (42.5)	407 (10.0)	-		926 (57.3)
Levosimendan, *n* (%)	73 (20.2)	109 (13.1)	148 (3.6)	-		234 (14.5)
PDEi, *n* (%)	-	2 (0.2)	-	-		48 (3.0)
Vasopressor (%)						
Epinephrine, *n* (%)	-	14 (1.7)	360 (8.9)	-		142 (8.8)
Norepinephrine, *n* (%)	-	45 (5.4)	770 (19.0)	-		164 (10.1)

IQR: interquartile range; SBP: systolic blood pressure; LVEF: left ventricular ejection fraction; RV: right ventricle; ACS: acute coronary syndrome; PDEi: phosphodiesterase inhibitors.

**Table 2:** Pharmacologic and hemodynamic properties of adrenergic agents.

Agents	Dose	Receptors	Inotropism	PVR	SVR
Adrenaline	0.05–0.1 µg/kg/min	α ++/β1 +++ (β1 = β2)	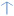 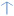 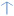	-	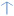
	High dose	A +++/β1 +++ (β1 = β2)	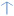 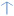 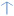 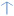	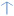	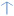 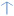
Noradrenaline	0.1–1.0 µg/kg/min	α +++/β1 + (β1 > β2)	-	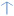 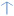	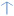 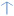 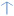
	High dose	α ++++/β1 ++ (β1 > β2)	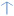	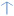 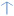 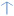	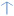 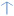 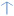 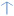
Dobutamine	2–10 µg/kg/min	β1 +++/β2 ++	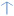 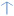 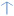	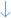	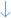
	>10 µg/kg/min	β1 ++++/β2 +++	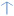 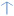 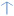	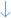 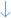	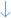 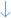
Dopamine	<3 µg/kg/min	D1 ++/β1 ++	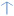	-	-
	3–5 µg/kg/min	D1 ++/β1 +++	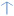 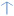	-	-
	>5 µg/kg/min	α +/β1 ++	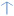 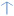	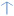	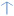
	>10 µg/kg/min	α +++/β1 ++	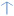	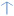 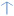	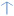 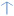

PVR: pulmonary vascular resistance; SVR: systemic vascular resistance. The increasing number of + indicates that the increasing number of receptors exibits a linear correlation with an upregulation or downregulation effect. The number of arrows pointing upwards (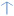) indicates a progressive increase in the effect, the number of arrows pointing downwards (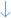) indicates a progressive decrease in the effect.

**Table 3:** Advantages and Disadvantages across different clinical setting.

Clinical Scenario	Advantages	Disadvantages
Cardiogenic Shock	Adrenaline: Systemic vasocostriction and inotropic effects while low/no effects on pulmonary resistance	↑ Myocardial oxygen demand Tachycardia
	Noradrenaline: Systemic effects on vasocostriction while not requiring myocardial oxygen consumption	↑ Afterload Low inotropic effects Peripheral ischemia
RV failure	Dobutamine: Inotropic effects with favorable RV/arterial coupling	Excessive drop of SVR Tachycardia (↑ ventricular response rate in patients with AF)
	Dopamine: At low doses, inotropic effects with neutral afterload	Tachycardia ↑ PVR (At high doses)
	Phosphodiesterase III inhibitors:Inotropic effects while reducing afterload (favorable RV/arterial coupling with particular effects on PVR drop)	Hypotension
	Levosimendan: Inotropic effects with vasodilatation and no tachycardia effect	Hypotension
LV Failure-AHF	Dobutamine Inotropic effects with mild vasodilatation (at low doses)	↑ Ventricular response rate in patients with AF
	Dopamine: Inotropic effects with neutral afterload (at low doses, vasodilatation by acting D1 receptors)	Tachycardia ↑ Afterload at high dose
	Phosphodiesterase III inhibitors: Inotropic effects with vasodilatation	Hypotension
	Levosimendan:Inotropic effects with reduced afterload, no arrhythmia effects, mitigating cardiac ischemia and tachycardia	Hypotension
LV-ADHF	Dobutamine: Inotropic effects while reducing PVR and SVR	Tachycardia (At a high dose, ventricular response rate, especially in AF with unfavorable effects)
	Dopamine:Low dose vasodilatation by acting D1 receptors	Unfavorable ventricular–arterial coupling (at high doses)
	Phosphodiesterase III inhibitors:Inotropic effects with vasodilatation; use in patients in pretreatment of BB therapy	Excessive/no tollarated vasodilatation effects (unfavorable effects especially in adavanced biventricular dysfunction)
	Levosimendan: Improved ventricular–arterial coupling Use in patients in pretreatment of BB therapy;	Longer half-life (unfavorable in patients who no longer need vasodilation)

SVR: systemic vascular resistance; RV: right ventricle; AF: atrial fibrillation; PVR: pulmonary vascular resistance: BB: beta-blockers. ↑: Increasing effect.

## Data Availability

Data sharing is not applicable to this article as no new data were created or analyzed in this study.
